# Mind and body connection in expert meditators: a computational study based on central and peripheral nervous system

**DOI:** 10.1186/s12906-024-04413-5

**Published:** 2024-03-07

**Authors:** Francesca Borghesi, Riccardo Cremascoli, Alice Chirico, Laura Bianchi, Amalia Di Moia, Lorenzo Priano, Alessandro Piedimonte, Alessandro Mauro, Pietro Cipresso

**Affiliations:** 1 Department of Psychology, University of Turin, Turin, Italy; 2 Istituto Auxologico Italiano, IRCCS, Unit of Neurology and Neurorehabilitation, San Giuseppe Hospital Piancavallo, Verbania, Italy; 3 Research Center in Communication Psychology, Universitá Cattolica del Sacro Cuore, Milan, Italy; 4 Department of Neurosciences Rita Levi Montalcini, University of Turin, Turin, Italy

**Keywords:** Affective States, Meditation, Psychometrics, Quantitative Psychology, Computational Psychometrics, Neurology, Neuroscience, Neurophysiology, Neuropsychology, Cognitive neuropsychology

## Abstract

A meditative ‘technique’ is conceived as a continuum of different affective states involving mind and body jointly. Meditative practices can involve cognitive effort (e.g., focused attention and open-minded techniques), as well as automatic and implicit practices (e.g., transcendental techniques). The NGALSO tantric self-healing meditation technique is a brief, comprehensive meditation technique relying on mind and body connection. In this study, we aimed to investigate the state and the trait neurophysiological correlates of NGALSO meditation practice. First, 19 EEG channels and a 3-lead ECG signal were recorded from 10 expert meditators (more than 7 years of daily meditation) and 10 healthy inexpert participants (controls) who underwent the same meditative procedure. The neuropsychological profiles of experts and controls were compared. Results showed that expert meditators had significantly higher power spectra on alpha, theta and beta, and a higher sympathetic tone with lower parasympathetic tone after meditation. Conversely, the control group had significantly less power spectra on alpha, theta and beta, and a higher parasympathetic tone with lower sympathetic tone after meditation. A machine learning approach also allowed us to classify experts vs. controls correctly by using only EEG Theta bands before or after meditation. ECG results allowed us to show a significantly higher effort by expert meditators vs. controls, thus suggesting that a higher effort is required for this meditation, in line with the principle ‘no pain, no gain’ in body and mind.

## Introduction

Meditation is a practice entailing an enhanced focus of the mind on a particular object, thought, or activity to achieve a mentally clear and emotionally calm state [1]. Travis and Shear [2] divided meditation practices into three categories: focused attention (FA) meditation, which entails voluntary and sustained attention on a chosen object; open monitoring (OM) meditation, which involves non-reactive monitoring of the moment-to-moment content of experience and transcendental meditation technique (TM), a process of automatic transcending based on mantra thinking [2]. A mantra is a word or phrase that is repeated, often as part of a meditation or prayer practice. From a psychological point of view, transcending is related to abandoning the here and now [3].

The two techniques (FA, OM) are characterized by focusing on individual control or effort, active mental processes that keep the brain engaged in a specific individual activity. Instead, TM is automatic because one uses the ‘‘natural tendency of the mind” to move from attention to mental silence. Each of the three meditation categories has distinct neurophysiological processes, attention management, and subject/object interactions [4]. Neurophysiological activation of the three types of meditation has been investigated deeply, focusing on central activations and peripheral activations such as heart rate variability or respiration. However, no study has directly compared these measures on FA, OM, and TM. In meditators, FA meditation is characterized by a central increment of beta (13–30 Hz) frequency power bands, OM monitoring by frontal theta bands (4–8 Hz) and TM by occipital alpha bands (8–13 Hz), analyzing only the difference in the effects of the meditation but without comparing them [5–9]. On the other hand, heart rate variability was mostly analyzed during the meditation, and in all techniques, there was an increase in the parasympathetic system and a decrease in the sympathetic one (Table 1) [10–13].

**Table 1:** Meditation type and neurophysiological activation

	**Focused attention (FA)**	**Open monitoring (OM)**	**Transcendental meditation (TM)**
**EEG (pr**e**-post meditation)**	BETA (central)	THETA (frontal)	ALPHA (occipital)
**ECG (during meditation)**	Increase parasympathetic activation, decrease sympathetic activation		

Generally, most studies consistently showed that meditators display an increased cerebral activation, especially in an increase in power of EEG theta bands, compared to controls (e.g., Zen meditation) in OM and TM techniques [14]. Other studies reported an increased basal cerebral activation in alpha and beta bands of meditators compared to controls (e.g., Vipassana meditation and Himalayan yoga) in FA techniques [15–17]. This suggested a trait (i.e., stable, individual) difference between meditators and controls (or non-meditators) in FA, OM and TM techniques [18–21]. However, it is still unexplored whether this discrepancy in terms of basal neurophysiological activation between expert meditators and non-expert meditators (control group) still holds for other more sophisticated meditative techniques [1]. This is the case for NGALSO meditative techniques—tantric self-healing practice—which can be described as a mixed meditation practice, integrating FA and TM meditation. NGALSO is a mixture of spiritual practice of active visualization technique and mantra meditation. This technique is assumed to heal the sicknesses of body and mind, not just the symptoms but the causes of individuals’ suffering. It is a meditation practice that transcends this life and helps to work for the benefit of all sentient beings. Lama Gangchen Rinpoche developed it at the beginning of the 1990s, but its origins can be traced back to Buddha’s teachings in the fourteenth century. NGALSO means ‘relaxation’ and can be divided into two syllables, Ngal and So. “Ngal” contains two noble truths: the truth of suffering and its causes. “So” contains the last two noble truths: the truth of the cessation of suffering and the path leading to the cessation. This meditation relies on imagery of facts and objects that evoke love, forgiveness, and compassion. Switching between visualizing sessions requires brief mantras. NGALSO meditation displays a dual nature because it alternates attentive imagery with brief mantras, becoming a blended approach between attentional and transcending meditation. Specifically, this meditation relies on imagery of facts and objects that evoke love, forgiveness, and compassion. Moreover, this kind of meditation is crucial in the aspects related to mentalization [22, 23].

The aim of NGALSO meditation is to induce transitions from normal consciousness to inner mindful attention states. It involves using various techniques, including breathwork, visualization, mantra chanting, and energy cultivation, to explore and integrate different aspects of the self [1].

Due to its nature, it could be used as a form of mind–body practice, as a complementary therapy [24–26]. In fact, tantric meditation aims to expand one's consciousness and awareness by exploring the interplay between the physical, mental, and spiritual realms. It encourages individuals to embrace all aspects of themselves, including their desires, emotions, and spirituality, fostering a sense of wholeness and connection [25, 26]. Through tantric practices, individuals can cultivate a deeper understanding of themselves, their patterns, and their reactions. This self-awareness can contribute to personal growth, healing, and the ability to make conscious choices in life. Tantra emphasizes the importance of conscious and authentic connections with others. Tantric meditation practices can enhance intimacy, communication, and connection within relationships, promoting empathy, compassion, and deeper emotional bonds [1, 2].

However, whether the mind and body connection is stable remains an open issue. That is, the short-term effect of NGLASO has never been explored before. NGLASO’s impact on individuals has not been studied at the state (i.e., contingent) level.

Our study aimed to explore and compare neurophysiological correlates of NGALSO meditation in expert meditators and a control group. To this end, we focused on and analyzed a timeframe of 2 min of eyes closed before and post-meditation. This allowed testing the impact of meditation at the trait, i.e., more stable label, as well as state, i.e., the short-term level, in the pre vs. post-meditation phase [18, 20].

We integrated EEG signals analysis and a computational approach (i) to test neurophysiological trait differences between meditators and controls localizing EEG significant channels and (ii) to show that we can predict accurately through machine learning if an undefined subject is a meditator or a control by relying exclusively on EEG bands, and, specifically, on theta bands.

## Materials and methods

### Participants

We recruited 10 expert meditators (EM) (*n* = 10; 7 females; mean ± SD meditation training: 32.2 ± 9.7 years) and 10 control group subjects without any prior meditation experience (CG) (*n* = 10; 3 females). Meditator participants were recruited from the people frequenting the Lama Gangchen Healing Center and with permission from the NGALSO Center. The inclusion criteria were senior practitioners (at least one long retreat with more than 7 years of daily practice) or teachers (more than 7 years of daily practice and have undergone several long retreats). The control subjects were people who had never meditated and had no intention of doing so or of embarking on a contemplative path. The groups did not differ significantly in age (EM = 48.89 ± 10,20; CG = 51 ± 13.19 years, t(14) = -0.362, *p* = 0.723, *d* = 0.18). Participants reported no psychiatric or neurological diagnoses. Each participant provided a written informed consent for study participation. Participants’ consent and all methods were carried out in accordance with the Helsinki Declaration.

### Procedure

Participants who met inclusion criteria were contacted via email and/or phone to organize a meeting in a standardized setting with a PC on a desk in front of the subject. Throughout each laboratory session, a neurophysiologist and a physician supported them. During their exposure to experimental stimuli, the experimenters were told to maintain a neutral attitude and tone of voice. When participants came to the Neurophysiology Laboratory, they were instructed to sit in front of a computer while the experimenters described the research objectives and the general function of the electrodes. After completing the informed permission form, participants were ready for the collection of physiological parameters.

The neurophysiological activation of the participants was recorded in a baseline resting state (pre-meditation condition). Then, the experimental session started, and physiological data were collected until the conclusion of the study. At the conclusion of the experimental session, the experimenters helped the participants remove all electrodes and patches and explained the scientific purpose of the experiment.

**Fig. 1:**
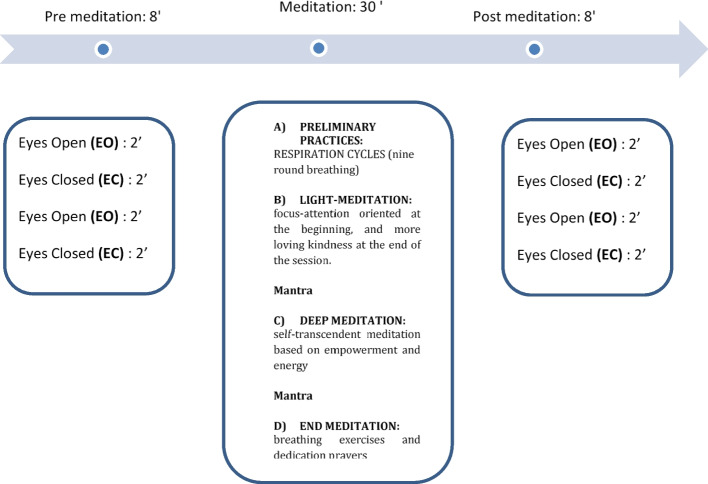
Meditation NGALSO program; EO = Eyes Open; EC = Eyes Closed

The meditation EEG protocol lasted about 46 min. It consisted of the following structure (See Fig. 1): pre-meditation eyes open (EO) and eyes closed (EC) for two minutes each, alternating twice – 8 min; NGALSO meditation 30 min; post-meditation EO and EC for two minutes each, alternating twice – 8 min. All NGALSO meditative states were in the eyes closed condition.

NGALSO practice consisted of different sessions, each associated with a specific meditative state (Fig. 1). A 30-min video by Lama Michel, the supervisor of the NGALSO center, provided instructions about the NGALSO complete meditation technique. Both groups (meditators and control subjects) watched it, projected on a TV screen in front of the subjects, to help them follow the correct sequence of the meditation protocol. Both groups listened to the same meditation video, keeping their eyes open to imitate and replicate the represented gesture. It is important to highlight that the meditation was considered active because the subjects (in both groups) were requested to interact actively with the narrative. Generally, meditation requires keeping the eyes closed and working on mental imagination. Keeping the meditation active while watching the video is a peculiar characteristic of NGALSO meditation. At the start of each meditative condition, time-locked markers indicating each session were displayed on the recording by a neurophysiologist trained in NGALSO protocol. The procedure allowed us to investigate each part of the NGALSO meditation technique and to know which parts were forgotten or missed by the meditators during the ECG and EEG recording sessions. The participants were explicitly instructed to relax and avoid meditating during the rest of the sessions.

### Materials

We used 19 EEG (electroencephalogram) channels with a longitudinal bipolar montage, which gives more localization than spectral measurements derived from monopolar reference schemes and is optimal for evaluating strongly localized low- to medium-amplitude waveforms [9, 27, 28]. Furthermore, the application of bipolar chains allows an approximation of the spatial derivative of the potential field, helping to pinpoint the locations of extreme electrical discharges within the EEG. By employing this method, we aimed to detect localized maxima (which, following convention, appear as minima due to the EEG's inverted nature) and potentially infer the underlying sources of these electrical activities [29].

**Fig. 2:**
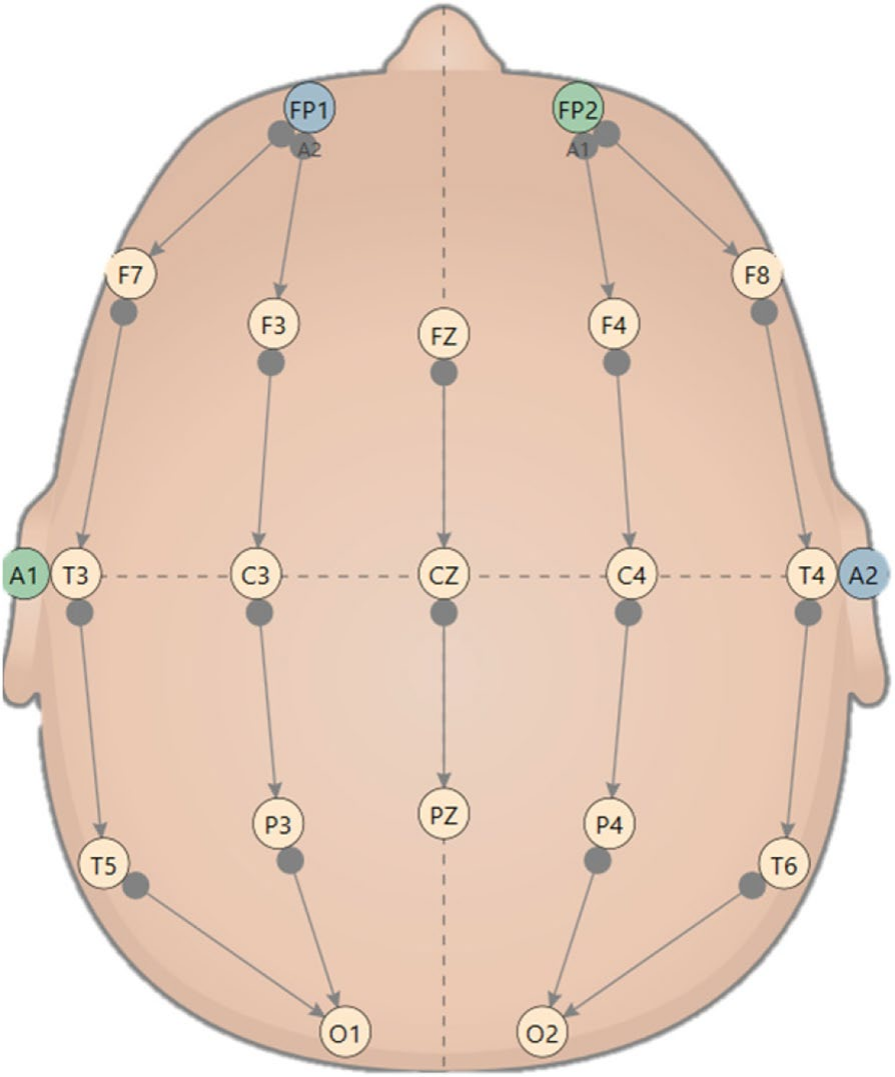
The bipolar-channels we used, following the International 10–20 locations

The data were bandpass filtered with a high bandpass filter of 0.5 to a low bandpass filter of 50 Hz. However, during the signal registration, a notch filter (50–60 Hz) was applied to remove electrical interference noise. Bipolar channel names based on the International 10–20 locations are: FP1-F7, FP1-F3, F7-T3, F3-C3, T3-T5, C3-P3, T5-O1, P3-O1 (left part); FP2-F4, FP2-F8, F4-C4, F8-T4, C4-P4, T4-T6, P4-O2, T6-O2 (right part); FZ-CZ, CZ-PZ (central part) (Fig. 2). The responses of the peripheral nervous system were measured using the specific EEG channels to catch a 3-lead ECG (electrocardiogram), amplified accordingly.

All the physiological signals were then processed with custom software developed using MATLAB 2022a (MathWorks, Inc.; Natick, MA) and SPSS 21 (Statistical Package for the Social Sciences–SPSS for Windows, Chicago, IL) with the use of Syntax to compute the statistical analyses. Every channel was synchronously acquired at 2048 Hz and exported at a 256 Hz sampling rate (256 records per second, one every 3.90625 ms).

The lab was equipped with two portable PCs, one for the EEG recording and delivering the stimuli (with a 120 Hz display) and the other for psychophysiological signal recording. Light and temperature sensors were used to monitor the room’s conditions to make interpretation relevant to users' actual affective state and avoid contamination.

### Signal processing

Collected EEG and ECG data were analyzed using MATLAB 2022a (MathWorks, Natick, MA) and EEG data were analyzed with Neurospectrum-5 software. For both signals, only the last 2 min of eyes closed before and after meditation (pre-post) were compared and analyzed. This choice depended on the desire for the cleanest possible pre-post measurement [30]. The meditation period was not an option as it would be full of movement artifacts because of the active meditation mode. Instead, the closed eyes pre and post allowed an objective measurement of the trait and state differences, both detected in the pre and maintained in the post-meditation.

### Mind measurements: EEG signal processing

**Fig. 3:**
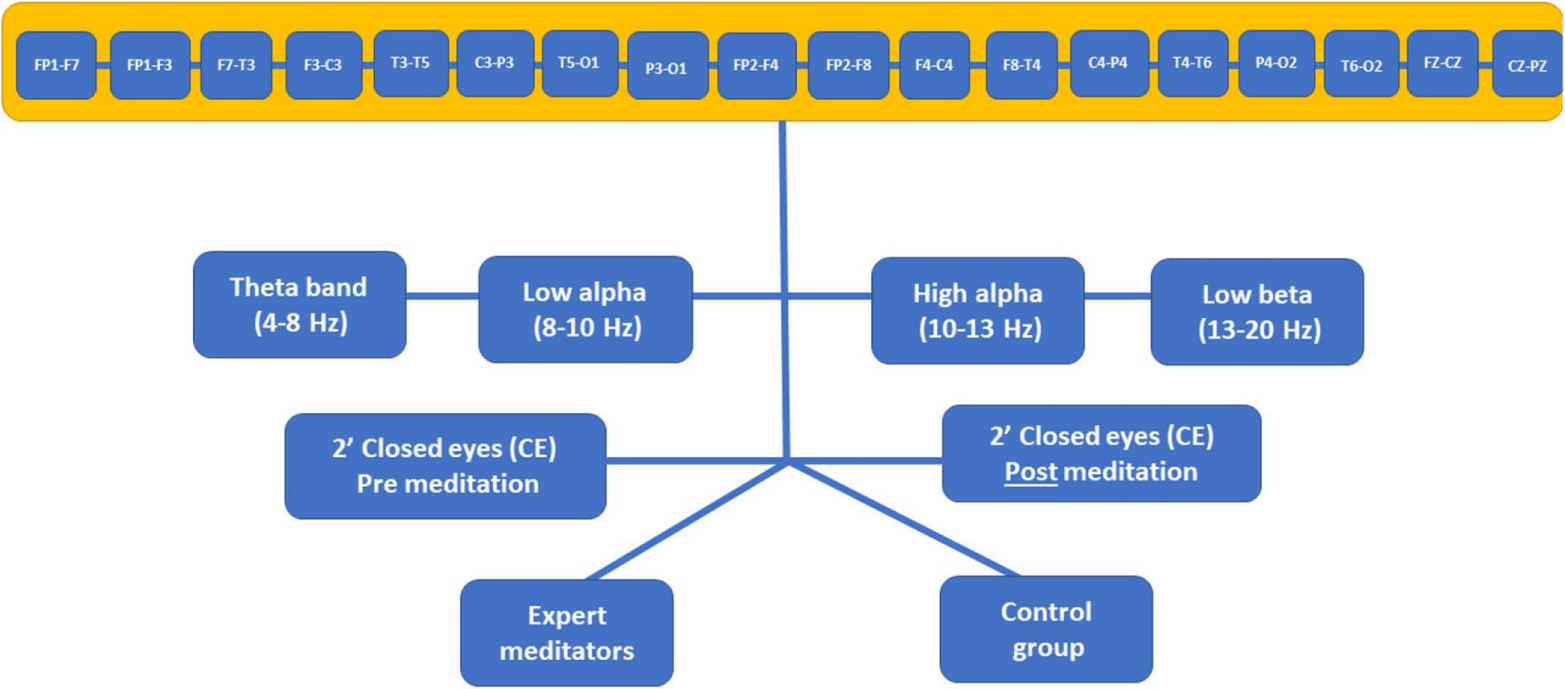
The extraction process for the spectral indexes from EEG

Matrixes have been computed to calculate power spectral EEG analysis bands, one per each bipolar-channel recorded. EEG signals were extensively worked to remove ocular artifacts, blinks and involuntary movements by visual inspection. Then we calculated beta EEG (e.g.,13–20 Hz) bands, high alpha EEG (e.g., 10–13 Hz) bands, and low alpha EEG (e.g., 8–10 Hz) bands and theta EEG (e.g., 4–8 Hz) bands. We did not include an analysis of gamma activity partly because of the known contribution of EMG activity to gamma activity in scalp recordings [31]. The mean was recorded for each participant at each electrode, both for baseline and post-meditation conditions. The data set was screened for normality and outliers before formal analysis. The mean regional data (frontal, temporal–central, posterior) for all four frequency bands ( beta, theta, high alpha and low alpha) were normally distributed for rest and meditation conditions (Fig. 3).

### Body measurements: ECG signal processing

Cardiovascular and respiratory activity were monitored to evaluate both voluntary and autonomic effects of respiration on heart rate, analyzing R-R intervals extracted from the electrocardiogram and respiration and their interaction. Following the guidelines of the Task Force of the European Society of Cardiology and the North American Society of Pacing and Electrophysiology, typical heart rate variability (HRV) spectral indexes were used to evaluate the autonomic nervous system response [32, 33].

As a temporal domain measure of heart rate variability, we calculated the mean RR index (ms), i.e., the mean of RR intervals and a longer mean RR interval means lower heart rate and higher parasympathetic cardiac activation; the root mean square of successive RR interval differences (RMSSD), which is a commonly used time-domain HRV parameter that captures the quick beat-to-beat changes in an RR interval; the mean heart rate (HR) interval and a higher heart rate are linked to higher sympathetic cardiac activation [34]; Baevsky’s stress index (SI) [35], reflecting cardiovascular system stress because high values of SI indicate reduced variability and high sympathetic cardiac activation.

As a non-linear domain, we calculated the Poincaré plot of short-term variability (SD1, ms) and the Poincaré plot of long-term variability (SD2, ms), both related to estimating the sympathovagal balance of the autonomic nervous system [36, 37]. SD1 is derived based on its dependency on the time-domain variable RMSSD. Instead, SD2 is derived based on its dependence on the time-domain variable standard deviation of RR intervals segments (SDNN), both described by Brennan and colleagues [38] and Cipresso and colleagues [39].

**Fig. 4:**
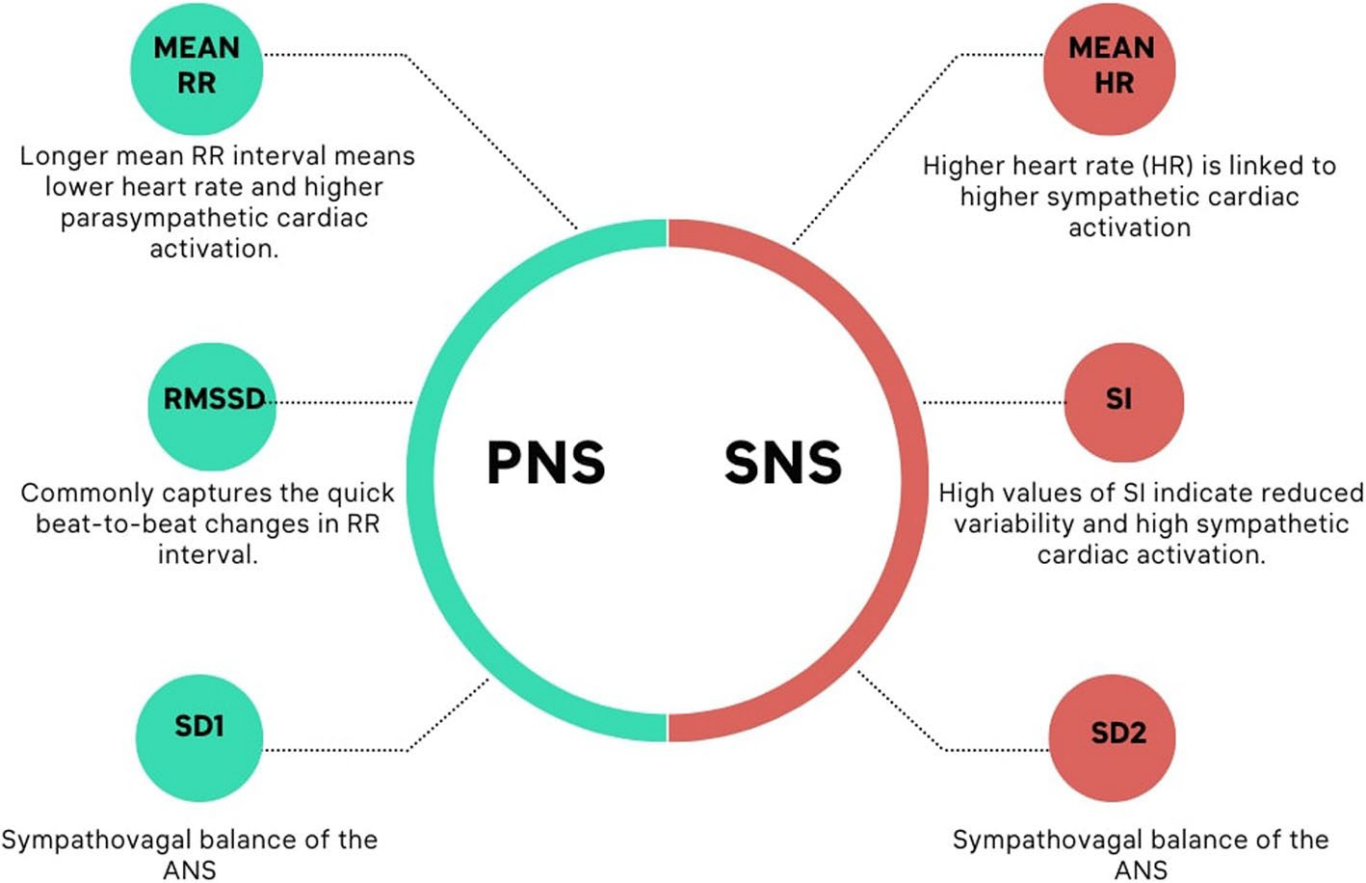
PNS and SNS index

We evaluated these 6 indexes to examine the short-term effects of meditation on sympathetic and parasympathetic tone. The parasympathetic nervous system (PNS) index was calculated based on the mean RR index, RMSSD, SD1, and the sympathetic nervous system (SNS) index was computed by the mean HR index, SI, SD2 (Fig. 4) [40, 41]. The calculation of the two indexes is a linear combination transformed into z-scores.

The activity of the parasympathetic nervous system on the heart is known to slow down the heart rate by increasing the time interval between successive heartbeats, enhance the respiratory sinus arrhythmia (RSA) component of HRV by increasing the rapid changes in RR intervals linked to breathing, and increase the proportion of rapid HRV fluctuations caused by RSA in relation to slower short-term fluctuations by decreasing the ratio between lower and higher frequency oscillations in the HRV time series. The PNS index highlights the short-term recovery using the parasympathetic tone in the SD1 nonlinear index combined with mean RR and RMSSD. The linear combination of these three indexes is computed in a z-score, so the PSN index units are also to be considered in the z-score. On the other hand, the activity of the sympathetic nervous system on the heart is known to increase heart rate, decrease the overall HRV by reducing the quick changes in RR intervals related to RSA, and increase the proportion of slower short-term fluctuations in the HRV data by increasing the ratio between lower and higher frequency oscillations in the HRV time series. The SNS index highlights the short-term stress using the sympathetic tone in the SD2 nonlinear index combined with mean HR and SI. The linear combination of these three indexes is computed in a z-score, so the SNS index units are also to be considered in the z-score.

### Statistical analysis

ECG and EEG data were analyzed using the statistical software SPSS, version 21 (Statistical Package for the Social Sciences—SPSS for Windows, Chicago, IL). A series of mixed-design ANOVAs with groups (meditators and control group) as between-subjects factors and time (pre-post meditation) as within-subjects factors were computed for EEG and ECG parameters. Partial eta square was calculated as a measure of effects for all measurements. ANOVAs were computed for EEG signals (Fig. 3) to test between effects regardless of being before or after the meditation, with the idea of testing the differences between expert meditators and the control group for each band in each location. On the other hand, the interaction effects between two main indexes of ECG signal, namely PNS index and SNS index, were computed to understand the impact of meditation on cardiovascular responses in both expert meditators and the control group.

### Computational analysis

EEG data were also used in multivariate computational analysis to classify the two groups using a supervised machine learning approach. The idea was to understand if, when using an EEG in a two-minute recording with closed eyes, we could tell if the brain ‘read’ is that of an expert meditator. Since previous studies focused on theta analysis, we computed these data segregated by bands, including theta, low-alpha, high-alpha, and beta.

Computational analyses were done using Python 3.4 with the Orange 3.3.5 data mining suite, accessible in the open-source code (https://github.com/biolab/orange3), from which it is possible to see all the algorithms used in the article. Leave-one-out cross-validation was done using the following methods [42, 43]: (1) logistic regression classification algorithm with ridge regularization; (2) random forest classification using an ensemble of decision trees; and (3) Naïve Bayes, a probabilistic classifier based on Bayes’ theorem. As stated before, all the algorithms used were available in the open-source code and documentation related to them can be found in the scikit-learn user guide, which provides a detailed explanation of all the algorithms used in the study, including rank calculation, classification tree, and learners (https://scikit-learn.org/stable/user_guide.html).

## Results

### EEG analysis

**Fig. 5:**
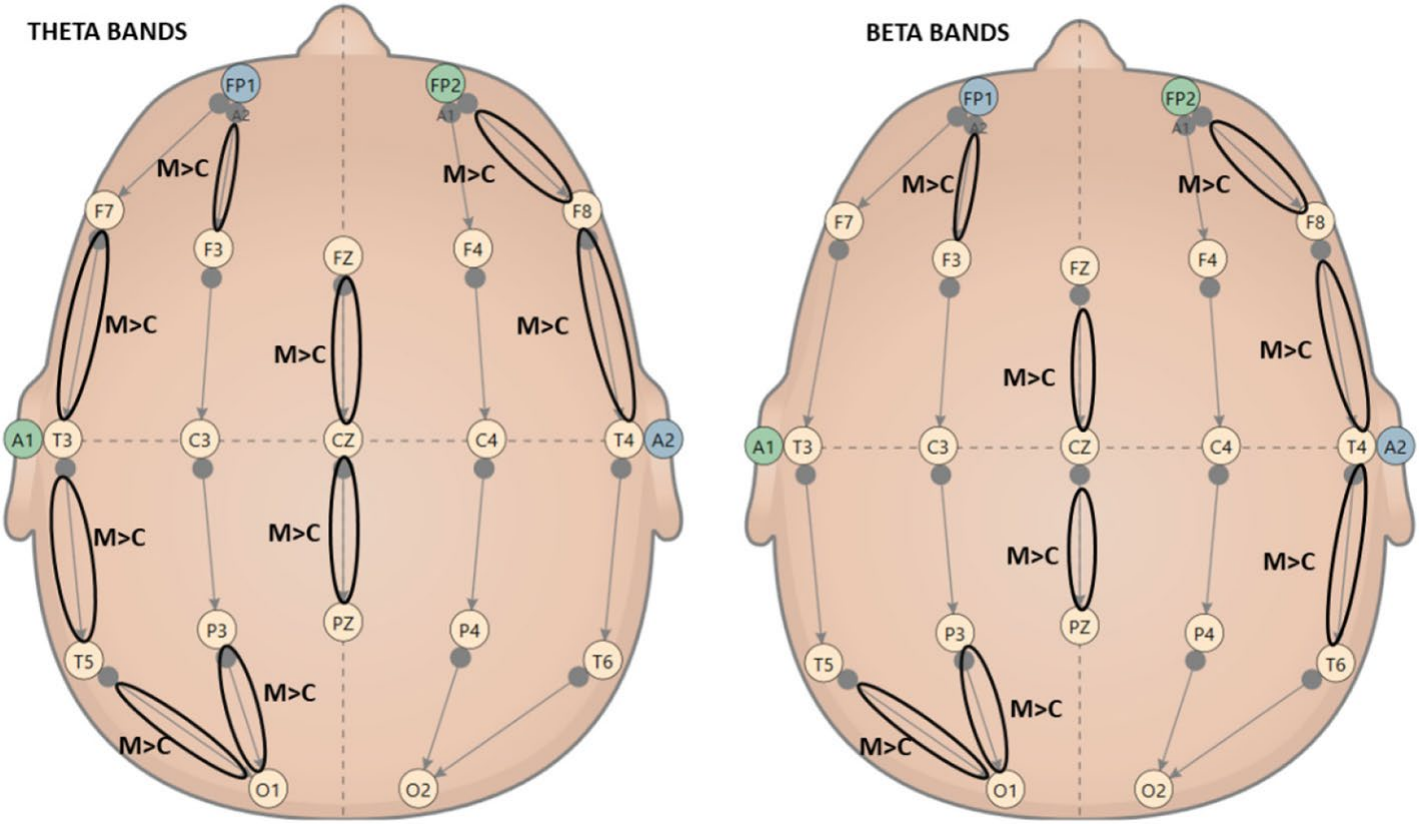
Statistically significant main effect of group in mixed ANOVA: Meditators (M) had higher power spectral than Controls (C) in Theta (left side) e Beta bands (right side)

**Fig. 6:**
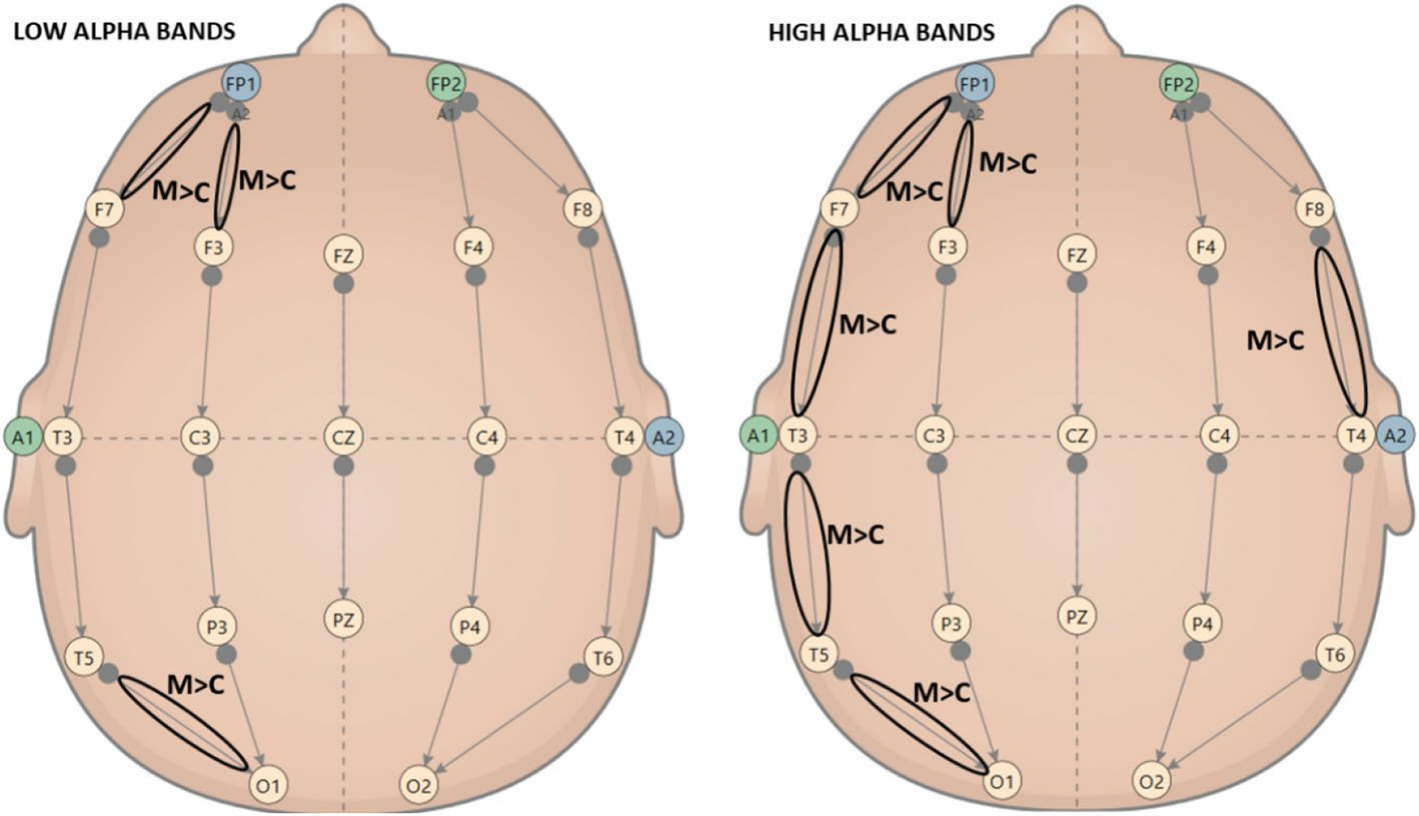
Statistically significant main between effect of group in mixed ANOVA: Meditators (M) had higher power spectral than Controls (C) in Low alpha (left side) e High alpha bands (right side)

A mixed ANOVA 2 (time: Pre vs. Post) × 2 (group: meditators vs. control group) was conducted on theta, low alpha, high alpha and beta frequency bands, comparing 10 meditators and 10 control subjects. Results showed a significant difference between the expert and control groups in terms of theta band, low alpha, high alpha and beta (Figs. 5 and 6), regardless of the timeframe. Partial eta square showed a high main effect of group, especially FP1-F7 (${\eta }_{p}^{2}$=0.981), FP2-F8 (${\eta }_{p}^{2}$=0.926), F7-T3 (${\eta }_{p}^{2}$=0.869) for theta bands, FP1-F7 (${\eta }_{p}^{2}$=0.817) for low alpha bands, T5-O1 (${\eta }_{p}^{2}$=0.833) for high alpha bands, FZ-PZ (${\eta }_{p}^{2}$=0.91) and T5-O1 (${\eta }_{p}^{2}$=0.826) for beta bands (Table 2). Here, we reported only significant differences for each channel, but in general, meditators always had the highest values compared to controls in all bands. Snedecor-Fisher F statistics accounted for dependencies from repeated measures and corrections in its related *p*-values were reported with the partial eta-squares [44].

**Table 2:** The main significant effects of “group” (meditators and control group) on physiological indexes (*p* < .05), with mean and standard deviation

		**Meditators**		**Control group**				
		**Pre-** **meditation**	**Post-** **meditation**	**Pre-** **meditation**	**Post-** **meditation**	**F** **(1,18)**	**Sign.of** **group effect** **(*p*-value)**	**pη**^**2**^
**Theta**	FP1-F7	0.501(0.211)	0.474 (0.310)	0.159 (0.075)	0.138 (0.061)	18.170	0.001	0.981
	P3-01	0.767 (0.775)	0.687 (0.667)	0.203 (0.146)	0.235 (0.163)	4.775	0.042	0.543
	FP2-F8	0.549 (0.352)	0.411 (0.216)	0.150 (0.081)	0.172 (0.163)	13.005	0.002	0.926
	F7-T3	0.696 (0.441)	0.707 (0.562)	0.190 (0.067)	0.184 (0.095)	10.640	0.004	0.869
	F8-T4	0.602 (0.648)	0.565 (0.531)	0.130 (0.065)	0.153 (0.072)	5.605	0.022	0.662
	T3-T5	1.427 (1.496)	1.465 (1.664)	0.291 (0.186)	0.282 (0.131)	5.365	0.030	0.592
	T5-01	0.542 (0.545)	0.535 (0.578)	0.089 (0.051)	0.096 (0.059)	6.134	0.022	0.662
	FZ-CZ	0.942(0.905)	0.760 (0.975)	0.163 (0.088)	0.160 (0.082)	5.552	0.030	0.606
	CZ-PZ	1.081 (1.210)	0.867 (1.240)	0.161 (0.095)	0.164(0.108)	4.465	0.049	0.516
**Low alpha**	T5-01	1.561 (1.728)	1.628(1.738)	0.460 (0.452)	0.37 (0.319)	4.475	0.049	0.517
	FP1-F7	0.787 (0.556)	0.816 (0.587)	0.250 (0.199)	0.224 (0.169)	9.014	0.008	0.817
	FP1-F3	0.446 (0.343)	0.419 (0.321)	0.151 (0.116)	0.130 (0.059)	7.478	0.014	0.734
**High alpha**	T5-01	0.554 (0.310)	0.576 (0.312)	0.209 (0.186)	0.250 (0.176)	9.584	0.006	0.833
	T3-T5	0.950 (0.607)	1.040 (0.681)	0.416 (0.420)	0.410 (0.230)	7.185	0.015	0.718
	F8-T4	0.398 (0.194)	0.413 (0.237)	0.209 (0.113)	0.242 (0.106)	6.129	0.023	0.655
	F7-T3	0.588 (0.466)	0.679 (0.521)	0.290 (0.215)	0.275 (0.172)	4.667	0.044	0.534
	FP1-F7	0.339 (0.291)	0.378 (0.294)	0.105 (0.049)	0.103 (0.054)	7.462	0.014	0.734
	FP1-F3	0.241 (0.151)	0.227 (0.183)	0.085 (0.041)	0.102 (0.069)	6.704	0.019	0.688
**Beta**	CZ-PZ	0.262 (0.138)	0.273 (0.167)	0.142 (0.089)	0.131 (0.074)	5.872	0.026	0.630
	FZ-PZ	0.280 (0.138)	0.269 (0.155)	0.111 (0.062)	0.105 (0.050)	12.184	0.003	0.910
	T5-O1	0.432 (0.337)	0.503 (0.447)	0.104 (0.063)	0.096 (0.039)	9.398	0.007	0.826
	T3-T5	1.473 (1.845)	1.769 (2.071)	0.350 (0.173)	0.306 (0.134)	4.807	0.042	0.546
	F8-T4	0.742 (0.574)	0.926 (0.836)	0.308 (0.303)	0.288 (0.219)	5.898	0.026	0.632
	P3-O1	0.343 (0.206)	0.397 (0.312)	0.167 (0.102)	0.162 (0.076)	5.707	0.028	0.618
	FP1-F3	0.558 (0.323)	0.537 (0.290)	0.167 (0.137)	0.301 (0.518)	5.327	0.033	0.589
	FP2-F8	0.727 (0.753)	0.727 (0.701)	0.178 (0.142)	0.287 (0.246)	4.457	0.049	0.515

**Fig. 7:**
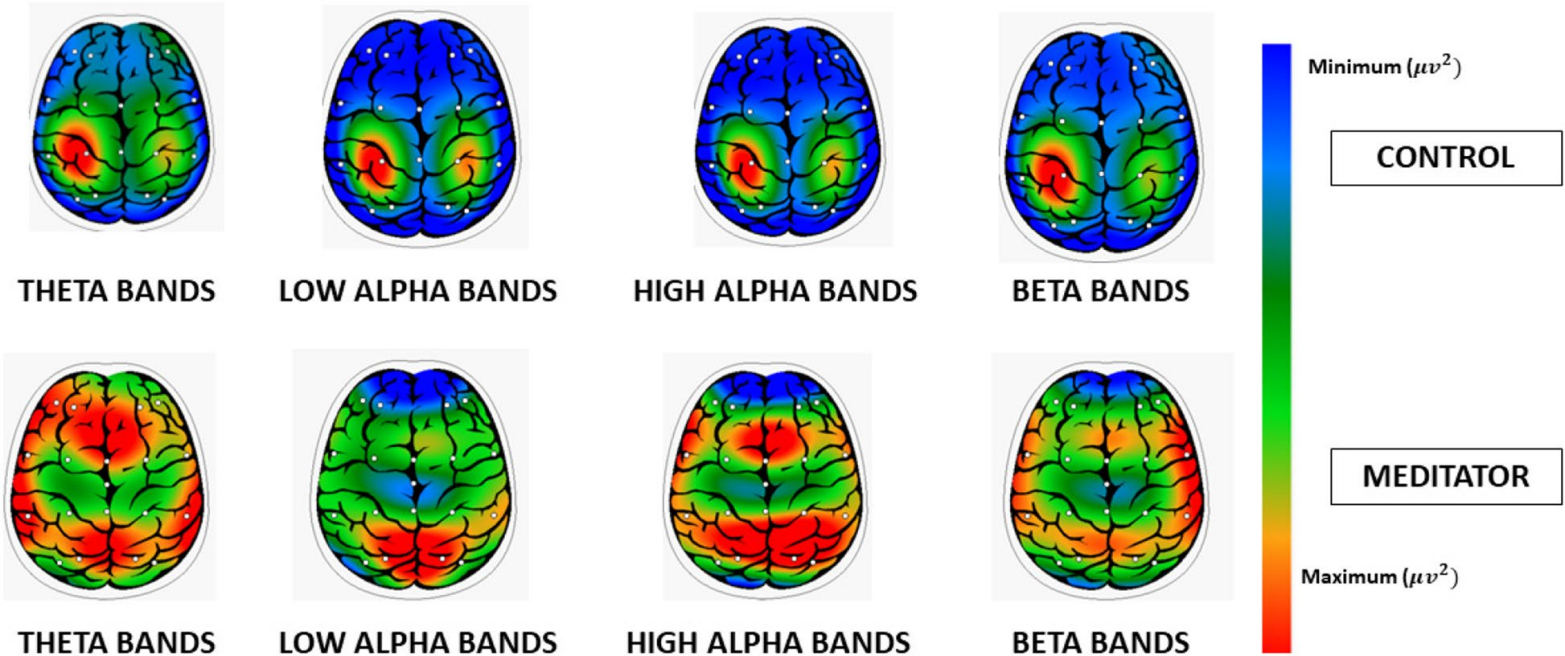
Power Spectral map bands comparison between Control vs. Meditator

**Fig. 8:**
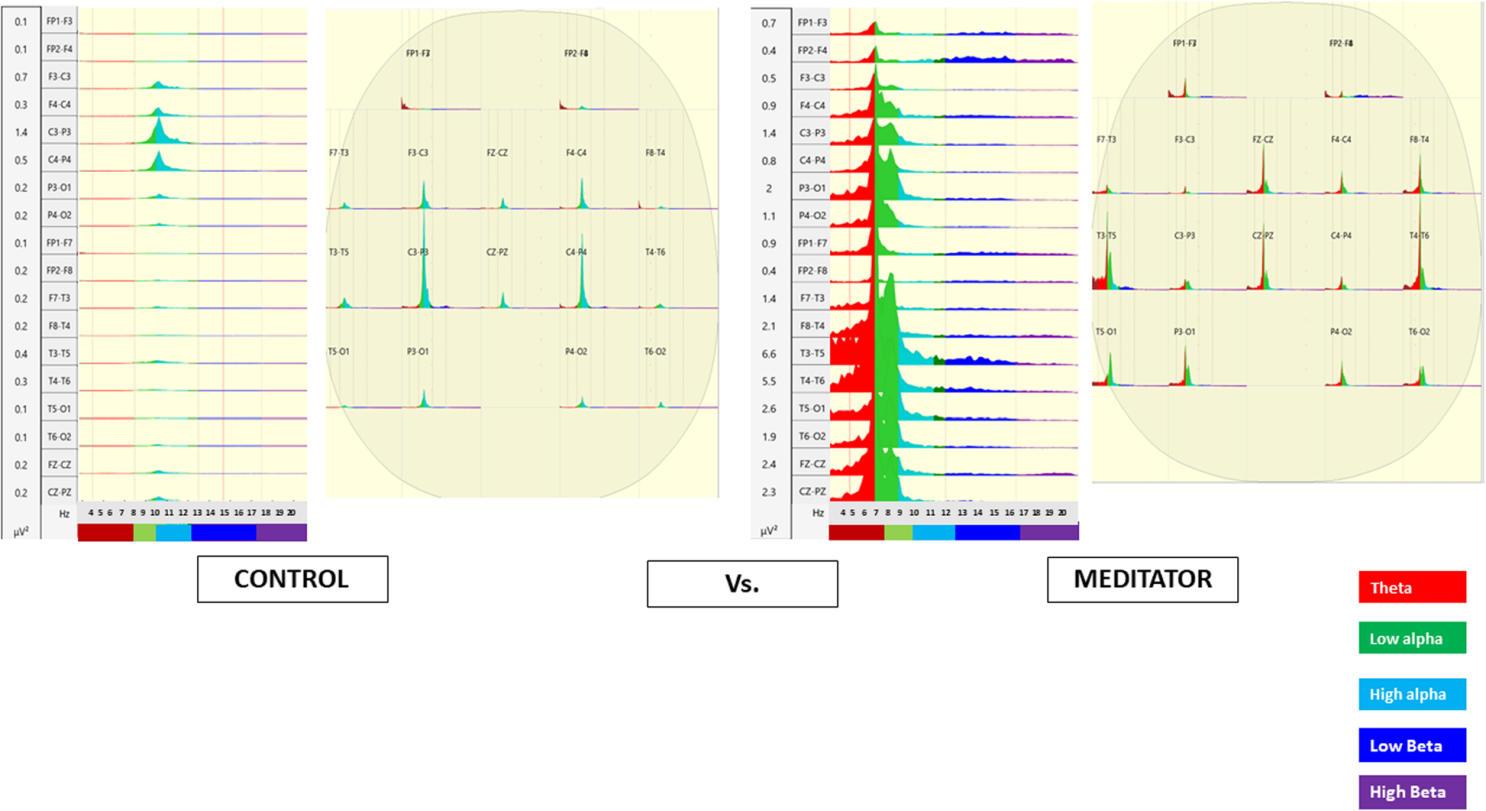
Power spectral graph between Control vs. Meditator

EEG analysis showed a significant main effect of the group on all bands considered. This effect was constant in pre and post-meditation; demonstrated meditators had substantial central activation differences: NGALSO meditation leaves a specific central activating imprint on meditators, who outperform controls in both pre and post-meditation Fig. 7 shows a comparison between a typical spectrum map of band frequency of meditator vs. control, and Fig. 8 a spectral graph divided in bands.

Moreover, the results showed that the frontal lobe is frequently involved in montage and has the most considerable effective size. The frontal lobe may play an important role in mental-effort work during meditation [45–48].

#### Computational analysis on EEG data

**Fig. 9:**
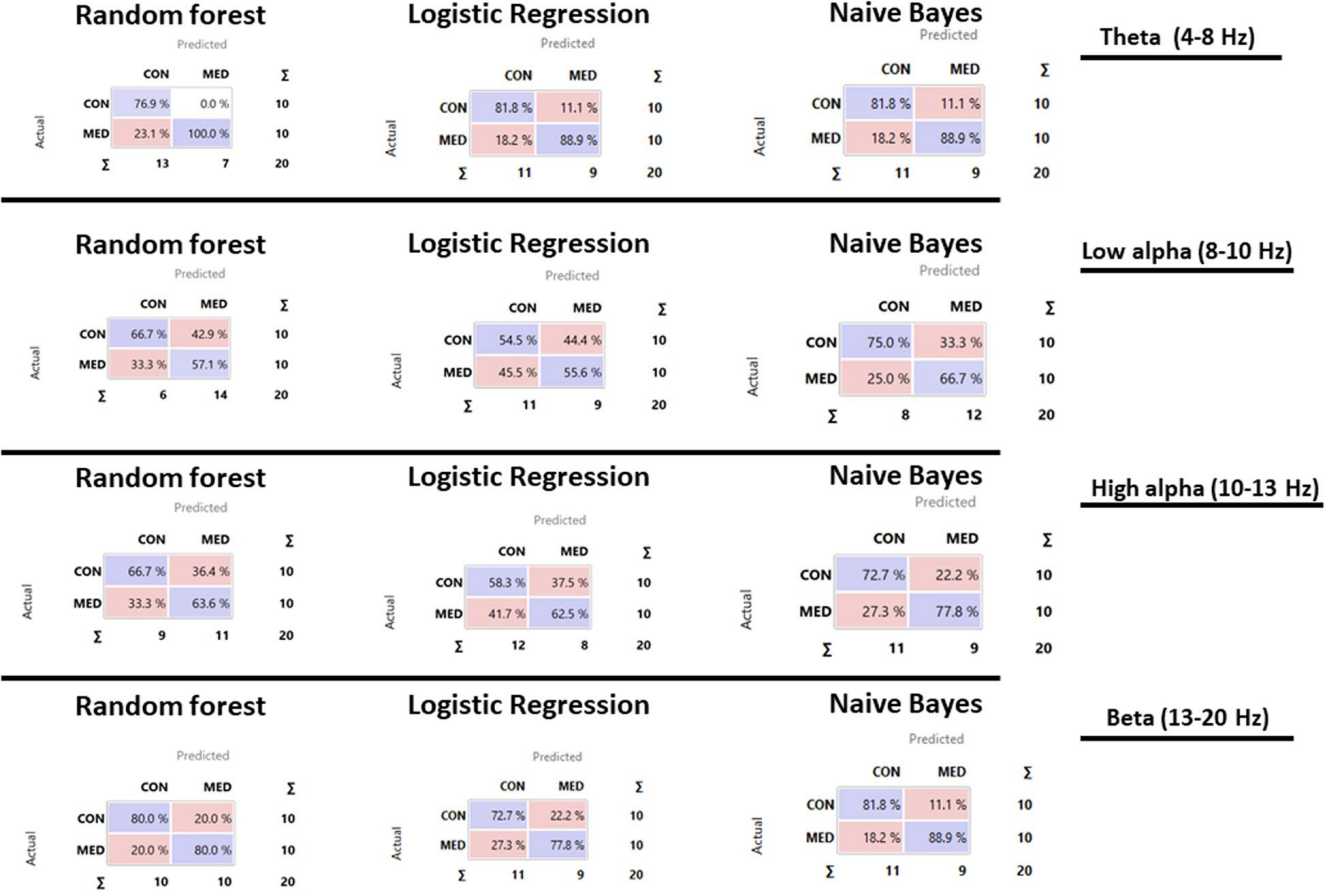
Confusion matrixes for the three classification methods. The diagonal values (i.e., purple values) represent the elements for which the predicted group is equal to the true group, while of-diagonal elements are those that are mislabeled by the classifier

In addition, nonlinear stochastic approximation multivariate methods confirmed the ANOVA results, showing an excellent capacity in discriminating meditators and controls. We conducted computational analyses using the leave-one-out cross-validation (LOOCV) method and results shown in Table 3 highlight a true positive rate of discriminating (sensitivity) between 80 and 100% for theta bands, while it ranged from 60 to 70% for low alpha, 60% to 80% for high alpha and 70% to 90% for beta. Interestingly, all algorithms, especially random forest for theta bands (83% discriminating capacity for control and 90% for meditators) showed that power bands had a higher capability of predicting meditator group or control group (Fig. 9).

**Table 3:** Leave-one-out cross-validation method: Three learning algorithms were compared, i.e., 1) Logistic Regression, 2) Naïve Bayes, 3) Random Forest. In the analysis, the classification learning algorithms were used for the classifications referring to the EEG

	**Model**	**AUC**	**CA**	**F1**	**Precision**	**Recall**
**Theta**	Logistic Regression	0,88	0,85	0,85	0,85	0,85
	Naive Bayes	0,97	0,85	0,85	0,85	0,85
	Random Forest	0,98	0,85	0,847	0,88	0,85
**Low alpha**	Logistic Regression	0,57	0,55	0,54	0,55	0,55
	Naive Bayes	0,75	0,70	0,69	0,70	0,70
	Random Forest	0,71	0,700	0,69	0,70	0,70
**High alpha**	Logistic Regression	0,60	0,60	0,59	0,60	0,60
	Naive Bayes	0,73	0,75	0,75	0,75	0,75
	Random Forest	0,82	0,80	0,80	0,80	0,80
**Beta**	Logistic Regression	0,73	0,75	0,74	0,75	0,75
	Naive Bayes	0,89	0,85	0,85	0,85	0,85
	Random Forest	0,89	0,85	0,85	0,80	0,80

*AUC* (Area under the ROC curve) is the area under the classic receiver-operating curve

*CA* (Classification accuracy) represents the proportion of the examples that were classified correctly

*F1* represents the weighted harmonic average of the precision and recall (defined below)

*Precision* represents a proportion of true positives among all the instances classified as positive. In our case, the proportion of a condition was identified correctly

*Recall* represents the proportion of true positives among the positive instances in our data

**Fig. 10:**
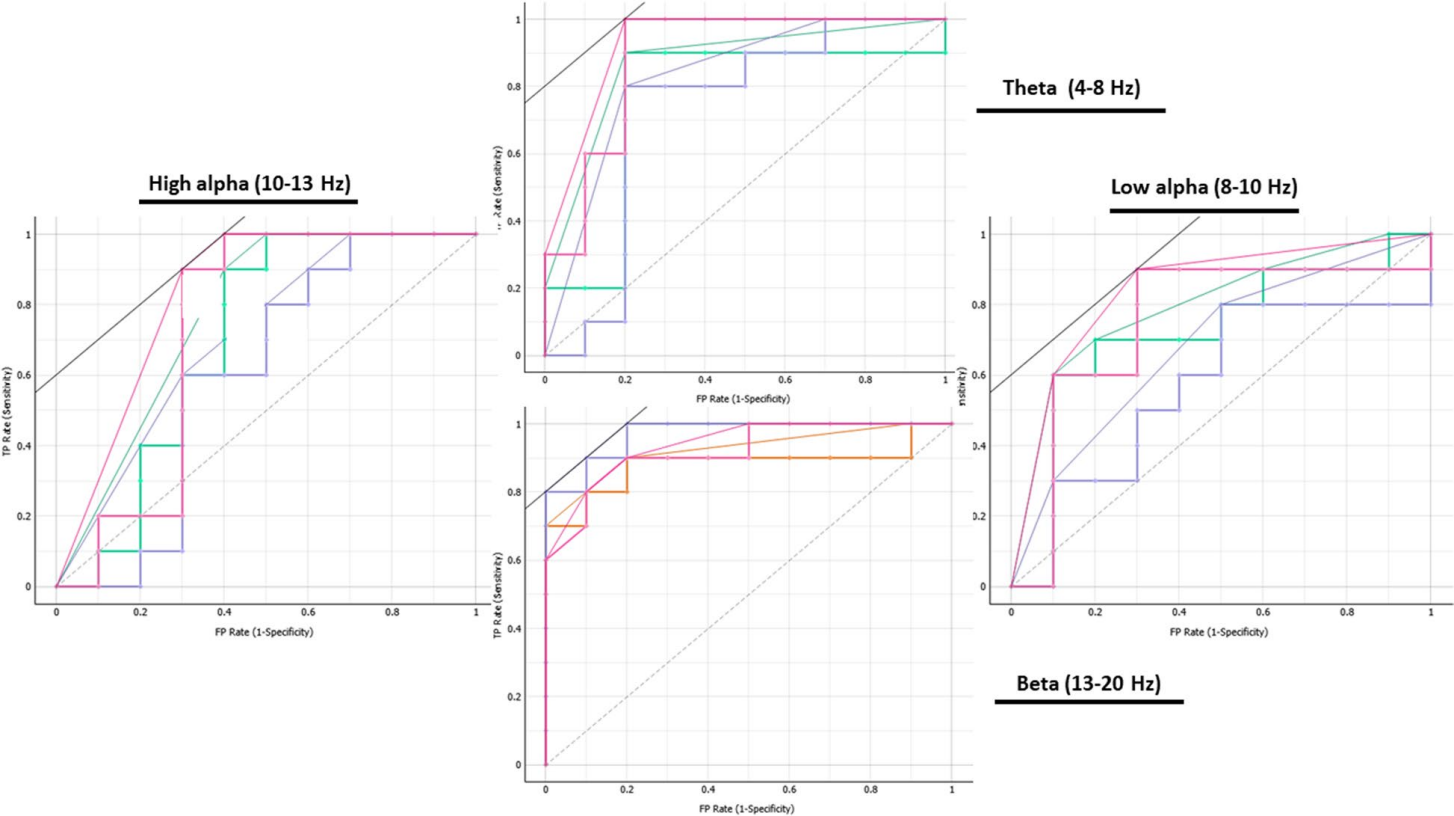
ROC analysis based on prediction of Meditators group: The area under the ROC curve represents accuracy (AUC) of each algorithm. The dotted line that goes from the point (0,0) to (1,1) represents the ROC curve for a random model: a model that predicts a 0 half of the time and a 1 half of the time, independently of its inputs. The pink line represents Naïve bayes logarithm, Violet one Logistic Regression logarithm, and Green one Random Forest logarithm

For the evaluation of different algorithm ROC or Receiver Operating Characteristic analysis was used. The curve plots a false positive rate on an x-axis (1-specificity; probability of False data points that are correctly classified) against a true positive rate on a y-axis (sensitivity; probability of True data points that are correctly classified). The closer the curve follows the left-hand border and then the top border of the ROC space, the more accurate the classifier (Fig. 10).

### ECG analysis

**Fig. 11:**
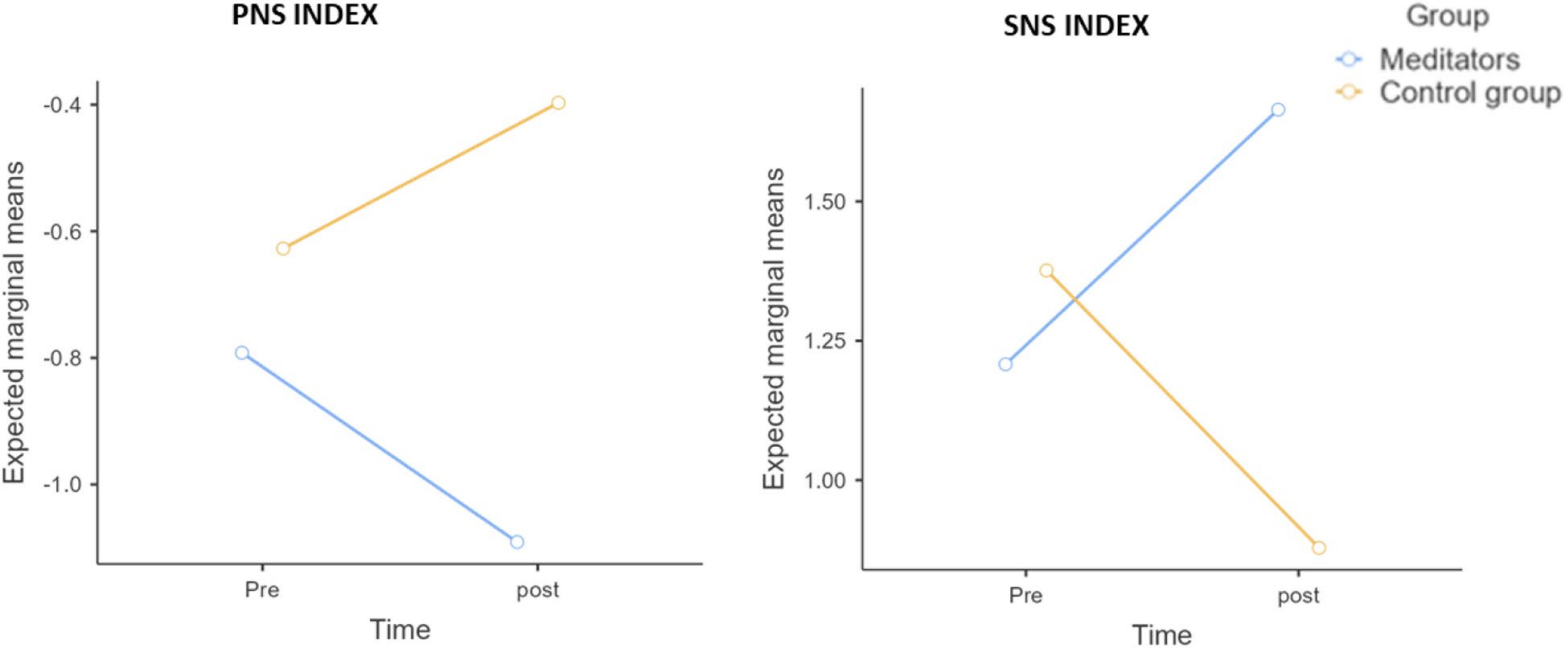
Parasympathetic vs. Sympathetic nervous system

Only *interaction effects* in a mixed ANOVA 2 time (Pre vs. Post) × 2 group (experts vs. controls) were statistically significant for *PNS index* F (1,18) = 5.803, *p* = 0.027, pη^2^ = 0.625 and SNS index F(1, 18) = 5.415, *p* = 0.032, pη^2^ = 0.596 (Fig. 11).

ECG analyses, instead, showed an opposite effect in post-meditation caused by the interaction effect between the two groups (expert meditators vs control subjects). The PNS index and SNS index, a mix of temporal and non-linear parameters, revealed how the effect of NGALSO meditation was significantly different between the two groups. The PNS index increased in the control group, while it decreased in meditators. On the other hand, SNS increased in meditators and decreased for the control group (Table 4).

**Table 4:** Descriptive statistics for PNS and SNS indexes of parasympathetic and sympathetic activity

	**PNS index**		**SNS index**	
**Group**	**Pre**	**Post**	**Pre**	**Post**
*Meditators*	-0.792 (0.721)	-1.090 (0.588)	1.210 (0.690)	1.660 (0.753)
*Control group*	-0.627 (1.030)	-0.397 (1.100)	1.380 (1.250)	0.879 (1.030)

## Discussion

Meditation is a complex neurophysiological concentration and mindfulness state. Also, given its multidimensional nature, a consensus on its operationalization as a complementary therapy still needs to be achieved. This issue, potentially, may have led to a limited number of studies comparing non-meditators and meditators. To address this issue, this research focused on a sophisticated active meditation technique, i.e., NGALSO, associated with focused attention (FA) and transcendental meditation (TM), and we compared expert meditators and control subjects at the trait and the state level. That is, first, basal neurophysiological profiles of meditators and non-meditators were compared. Then, the short-term effects of NGALSO were measured in meditators and non-meditators, using EEG analysis combined with a machine learning approach. Based on previous studies on the neurophysiological activation of meditators and non-meditators, a difference in terms of central and peripheral effects was expected.

Overall results suggested that meditation intervention had a significant main effect of group on EEG spectra and a large intersection effect on ECG parameters. The main effect of group was detected at the trait level, since it was found that meditators and control subjects significantly differed in spectral activation in the pre-meditation phase. This result was also found in the short-term run, in the post-meditation phase at the state level. This confirmed that expert meditators have the highest power of spectral analysis on all the interested bands (alfa-theta- beta). Previous studies demonstrated specific activation patterns compared to control groups based on meditation type (AF, OM, or TM) (4–9). In meditators, FA meditation was characterized by a central increment of beta (13–30 Hz) frequency power bands, OM monitoring by frontal theta bands (4–8 Hz) and TM by occipital alpha bands (8–13 Hz), compared to pre-post meditation power bands. NGALSO is a mixture of AF and TM meditation. For this reason, the main spectral difference for all bands considered is due to its double nature (mix of AF and TM). High alpha bands for the mantra are typical of TM, and high beta bands for the visualization part are typical of AF. The theta bands reflect mental concentration as well as a meditative state. They are known to be linked to states of self-awareness with disciplined regulation of attention, breath, and bodily posture.

Nonlinear stochastic approximation machine learning methods confirmed these effects. Random forest, logistic regression and Naïve Bayes algorithms demonstrated that unique power frequency bands could predict and discriminate meditator traits compared to the control group. Theta bands had the highest true positive rate for distinguishing controls from meditators (80% of instances classified as “control” were in the control class and 100% for meditators). These results are in line with previous findings on the role of alpha and beta bands [5, 49] and outlined a novel, unique pattern for theta bands, which can be explained as a typical correlate of NGALSO components of enhanced self-awareness and attention. Furthermore, other peripheral state changes emerged in meditators vs controls after meditation. PNS and SNS indexes had different activation patterns compared to meditators and controls. In meditators, the SNS index increased, while in controls, it decreased. In contrast, the PNS index increased in controls and decreased for meditators. Indeed, on a peripheral level, the control subjects felt more relaxed and calm with higher activation of the parasympathetic tone (PNS). On the other hand, for the expert meditators, meditation was a cognitive and effort task with actively focused concentration, resulting in a larger sympathetic tone activation (SNS). In fact, PNS activity is known to decrease heart rate and increase heart rate variability in neutral or calm contexts, whereas SNS increases HR and decreases HRV during stressful or challenging situations [50].

Meditators, following the practice of NGALSO meditation, demonstrated heightened sympathetic responses in the post-meditation phase. This augmented sympathetic activity can be attributed to the deliberate and focused attention required during NGALSO practice. NGALSO experts engage in a purposeful cognitive effort, intricately involving both body and mind, aiming to enhance their emotional and cognitive self-awareness. This intentional cognitive engagement is notably reflected in the observed increase in sympathetic activation post-meditation.

Conversely, individuals in the control group, despite engaging in meditation practices, exhibit a contrasting response characterized by a more tranquil and deactivating effect. Their naive approach to meditation leads to an elevation in parasympathetic tone post-practice. Unlike NGALSO experts who actively channel their cognitive faculties during meditation, naive meditators often experience a relaxation-oriented response, contributing to the observed rise in parasympathetic tone [50–53].

Finally, our results are specific to the two minutes before and after meditation, which means that meditators have unique central waves and peripheral parameters compared to controls. This activation shows how beneficial deep meditation could be to its practitioners. Tantric meditation often emphasizes the integration of the mind, body, and spirit [14, 25, 54]. It encourages individuals to embrace their physical sensations, bringing mindfulness to bodily experiences and sensations and promoting a sense of embodied presence. Research suggests that meditation may help alleviate symptoms of anxiety and depression. It can give individuals a sense of calmness, improve emotional well-being, and promote a more positive outlook on life [13, 19, 55–58]. Meditation techniques, such as tantric meditation, have shown promise in helping individuals cope with chronic pain. By focusing on the present moment and accepting sensations without judgment, individuals may experience reduced pain intensity and an improved ability to manage discomfort [51–53, 59–62].

However, a recent study highlighted the role of mental flexibility in meditation [63–65]. A future challenge of this study could effectively consider a subjective or objective way to measure mental flexibility to be assessed as integration. To the best of our knowledge, no current research on meditation has addressed specific mental processes involved in meditation effectiveness, in particular, mental flexibility.

Limitations of the study include the small sample size of 10 subjects per group, with a gender imbalance (7 Female for meditators vs. 3 female in the control group). However, gender inserted as a covariate in a mixed ANOVA was not significant. Huge effect sizes achieved (a partial eta-squared higher than 0.515) granted a statistical power higher than 99.98%; however, future studies should replicate this study and/or include a larger sample size. Another potential limitation to be addressed in future studies is the link between behavioral/psychological measures and neurophysiological correlates. This relationship could shed new light, above all in studying affect dynamics in relation to mental flexibility [66–70].

## Conclusions

The original contribution of this work concerns the neurophysiological correlates of active meditation, i.e., NGALSO, in expert meditators and control groups, measuring central and peripheral change effects. For meditators, meditation produced an enhanced cognitive effort (vs. controls), stimulating the sympathetic nervous system and altering their brain frequency spectra over time, especially alpha, beta, and theta bands. On the other hand, for controls, meditation emerged to be soothing and restorative for the mind and body, mainly activating the parasympathetic nervous system. Finally, this is the first study in which key differences between NGALSO meditators and controls were derived and predicted from their neurophysiological profile, thus suggesting that the impact of this meditative technique should be investigated in the long term, since these results showed that it could shape the way individuals’ brains work in a stable and consistent way.

## Data Availability

The datasets used and/or analyzed during the current study are available from the corresponding author upon reasonable request.
